# Fast three-dimensional black-blood MR imaging for carotid artery intra-plaque haemorrhage using DANTE-prepared FLASH (3D-DASH)

**DOI:** 10.1186/1532-429X-16-S1-O75

**Published:** 2014-01-16

**Authors:** Linqing Li, Luca Biasiolli, Joshua T Chai, Matthew D Robson, Robin P Choudhury, Ashok Handa, Peter Jezzard

**Affiliations:** 1 Department of Clinical Neurosciences, University of Oxford, Oxford, UK; 2 Department of Medicine, University of Oxford, Oxford, UK; 3 Acute Vascular Imaging Centre, University of Oxford, Oxford, UK; 4 Department of Surgery, University of Oxford, Oxford, UK

## Background

DANTE (Delays Alternating with Nutation for Tailored Excitation) pulse trains are a rapid series of low flip angle RF pulses interspersed with gradients. We have previously demonstrated that when using DANTE pulse trains as a preparation module prior to imaging readout, the longitudinal magnetization of flowing spins is substantially attenuated, whereas the longitudinal magnetization of static tissue/fluid is mostly preserved [1]. In this study we introduce a new DANTE-prepared 3D FLASH T1 weighted (T1w) sequence (denoted '3D-DASH')[2] that is able to generate 0.6 mm isotropic resolution images with an average imaging speed better than 2 sec/slice.

## Methods

6 healthy volunteers (males, 24 to 35 years) underwent (i) DIR (double inversion recovery)-prepared 2D-TSE, (ii) 3D-DASH and (iii) comparison MSDE prepared FLASH, 3D-MERGE, imaging[3]. 4 symptomatic patients (age range, 54-86) scheduled for carotid endarterectomy (> 70% stenosis measured by ultrasound) underwent the same vessel wall imaging protocol. Written informed consent was obtained from all subjects. All scans were acquired using a 3T Siemens Verio scanner. A pair of dual-channel surface coils (Machnet, The Netherlands) were used. Cardiac gating was used for comparison DIR-prepared black blood scans. Protocol: axial imaging acquisition, identical 3D FLASH readout sequences for 3D-DASH and 3D-MERGE, FOV = 150 × 150 mm, matrix size 256 × 252, interpolated to 512 × 512, partition thickness = 0.6 mm, Number of averages = 2, iPat = 2, slices = 128, FLASH flip angle α = 10°, slice resolution = 63%, phase and slice partial FT = 6/8, Fat suppression = water excitation-fast, TRinternal = 10 ms, BW = 130 Hz/pixel, resolution = 0.6 mm isotropic. Parameters for the DANTE module: flip angle (FA) α = 15°; Number of pulses Np = 150; time duration between DANTE pulses, tD = 1 ms; Gx, y, z = 20 mT/m; gradient duration≈1 ms.

## Results

**Figure 1:**
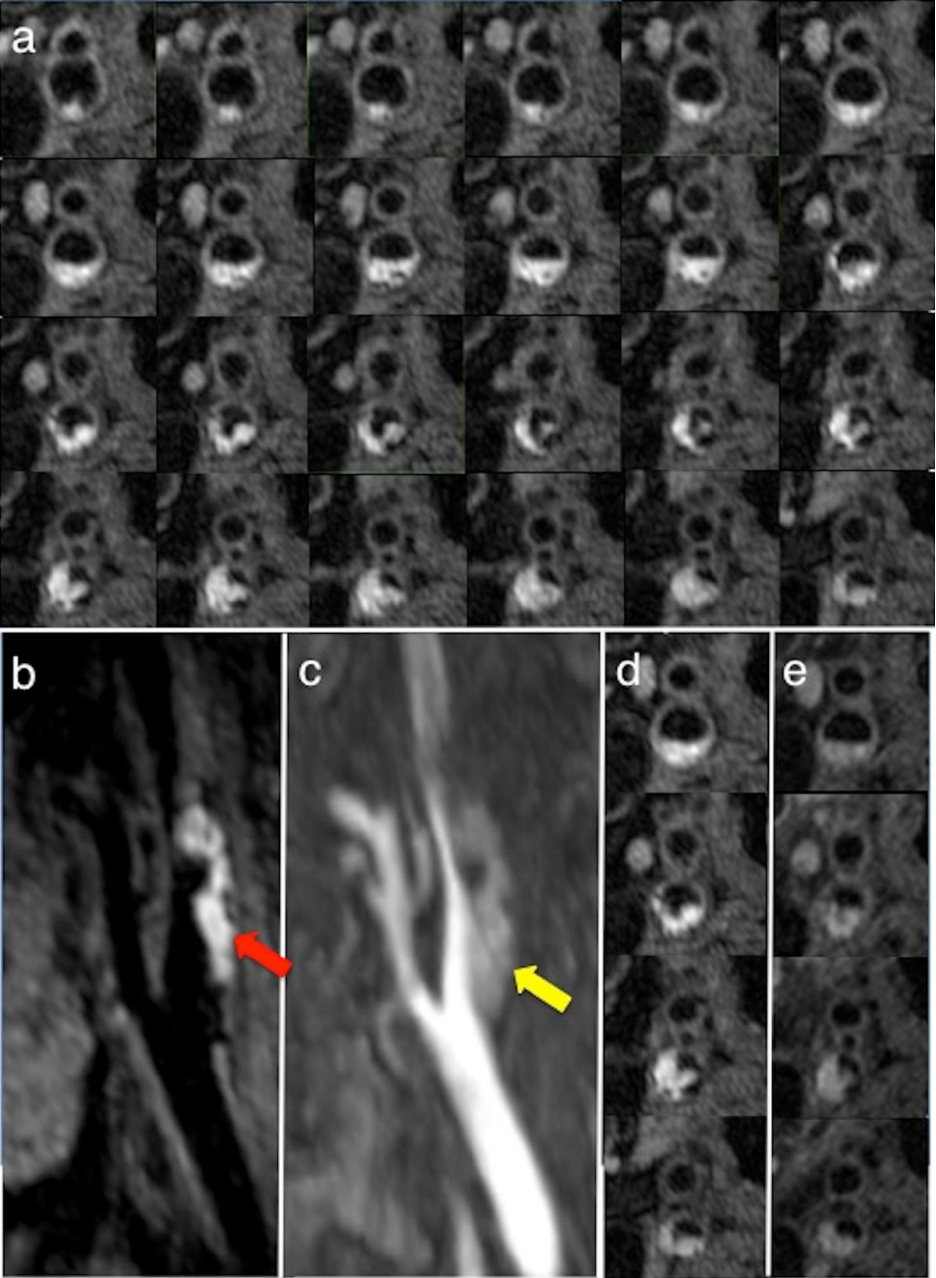
**T1w 3D-DASH images obtained from a patient with intra-plaque haemorrhage**. a) 24 contiguous-slice whole plaque coverage from 3D-DASH images with isotropic 0.6 mm resolution. b) 3D-MPR sagittal view of the left carotid arteries reconstructed from the full 128-slice 3D-DASH dataset. c) 3D-MPR sagittal view reconstructed from the 3D-TOF data for comparison. d) Axial view slices of 3D-DASH and e) DIR-prepared 2D-TSE images taken from the same slice positions for direct comparison.

**Figure 2:**
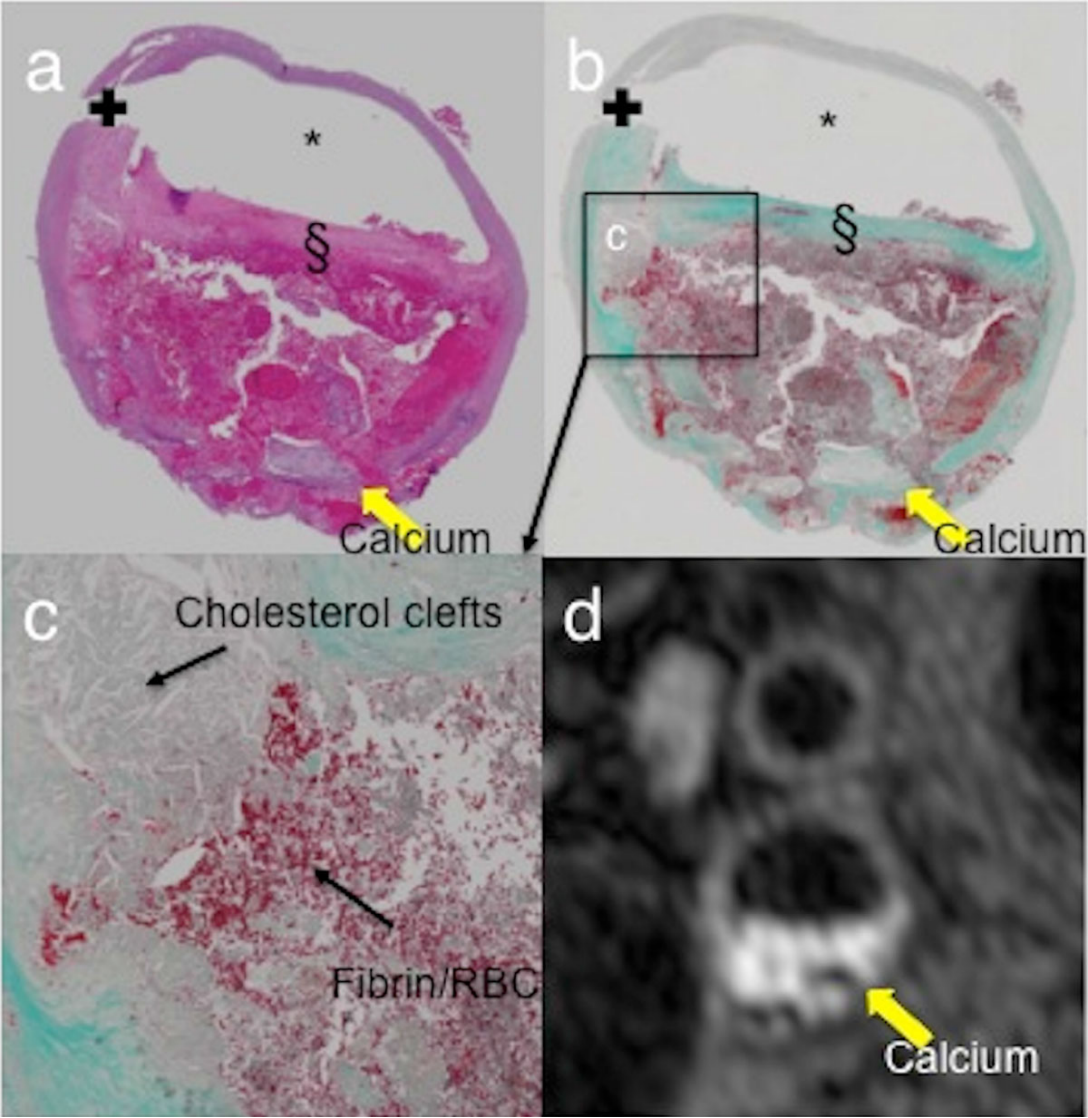
**This histology figure confirms the presence of intra-plaque haemorrhage of the patient plaque in Fig. 4**. a) Hematoxylin and eosin stain(H&E). * lumen; § fibrous cap; +surgical artefacts. b) Maisson's trichrome staining of serial 5 μm plaque sections. These images show a large lipid-rich necrotic core (LRNC) within an eccentric carotid atherosclerotic plaque with recent intra-plaque haemorrhage (IPH). c) Fibrin (in blood) stained bright pink in H&E and bright red in trichrome. Background lipid core seen as cholesterol clefts. Collagen stained green in trichrome. Calcification stained blue by haematoxylin and remained pale/unstained in trichrome. d) Image taken from section location 6 mm above bifurcation.

Examples of the T1w image quality for the 3D-DASH sequence with 0.6 mm isotropic resolution versus the gold standard single-slice DIR-TSE sequence with slice thickness 2 mm are shown in Figure 1. The 3D-DASH scan acquisition time was 198 seconds, with > 6 cm coverage (128 slices). The hyper-intense signal on the T1w images indicates the presence of fresh intra-plaque haemorrhage (IPH) confirmed by histological examination shown in Figure 2. Compared with the current best 3D black blood (BB) technique (results were not shown), 3D-MERGE, 3D-DASH allows 75%-100% improvement in contrast-to-noise efficiency, CNReff.

## Conclusions

3D-DASH is a promising new sequence for 3D isotropic (0.6 mm) fast black-blood T1 weighted imaging of the carotid arteries with high sensitivity to intra-plaque haemorrhage.

## Funding

We thank the NIHR Oxford Biomedical Research Centre, BHF, and Dunhill Medical Trust for grant funding.

